# Impact of Treatment Modalities on Locally Advanced Gastric Cancer—Real-World Data

**DOI:** 10.3390/curroncol32080463

**Published:** 2025-08-16

**Authors:** Esma Uguztemur, Banu Oztürk

**Affiliations:** 1 Adıyaman Training and Research Hospital Medical Oncology Department, Adiyaman 02040, Turkey; 2 Antalya Training and Research Hospital Medical Oncology Department, Antalya 07100, Turkey; banu.ozturk@saglik.gov.tr

**Keywords:** gastric cancer, neoadjuvant therapy, adjuvant therapy, survival, chemotherapy, surgical margin

## Abstract

The optimal sequencing of chemotherapy in locally advanced gastric cancer (LAGC) remains controversial. This study aimed to compare survival outcomes between adjuvant (ACT) and neoadjuvant (NACT) chemotherapy and to identify clinicopathological factors associated with progression-free survival (PFS) and overall survival (OS) in a real-world setting. Methods: We retrospectively analyzed 103 patients with non-metastatic gastric cancer treated between 2014 and 2024. Patients were categorized into ACT (*n* = 56) and NACT (*n* = 47) groups. Kaplan–Meier and Cox regression analyses were used to assess survival outcomes and prognostic factors. Results: The NACT group was younger and had more proximal tumors. Median OS was 48.7 months in the ACT group versus 17.7 months in the NACT group (*p* = 0.048). Median PFS was not reached in the ACT group and was 15.6 months in the NACT group (*p* = 0.008). Negative surgical margin status was independently associated with improved survival, whereas age was an independent negative prognostic factor for OS. No significant associations were found between OS or PFS and histologic subtype, lymphovascular invasion, perineural invasion, gender, D2 dissection, or type of surgery. Notably, 21% of NACT patients did not proceed to surgery due to progression, treatment intolerance, or refusal. Conclusion: Although ACT was associated with longer PFS and OS in this cohort, these differences are most likely explained by baseline imbalances, patient selection factors, and survivorship bias rather than the timing of chemotherapy itself. These findings highlight the importance of careful patient selection for NACT and underscore the need for prospective, randomized studies to define optimal sequencing strategies in LAGC. Our study contributes descriptive, real-world data rather than definitive evidence of treatment superiority.

## Simple Summary

Gastric cancer is a serious disease that often requires both surgery and chemotherapy to improve survival. However, there is ongoing debate about the optimal timing of chemotherapy—before surgery (neoadjuvant treatment) or after surgery (adjuvant treatment). In this study, we reviewed the medical records of 103 patients with stomach cancer that had not spread to other parts of the body. We compared the observed survival outcomes between those who received chemotherapy before surgery and those who received it afterward. Patients who received chemotherapy after surgery had longer observed survival than those who received it before surgery. This difference may be partly explained by the fact that a substantial proportion of patients starting treatment before surgery did not ultimately undergo surgery, often because their disease progressed or they experienced treatment-related side effects. Our findings highlight the importance of careful patient selection for neoadjuvant chemotherapy and indicate the need for further studies to guide treatment planning in gastric cancer.

## 1. Introduction

Gastric cancer is the fifth most prevalent malignancy worldwide and the fifth most common cause of cancer-related deaths [1]. Despite advances in diagnosis and treatment, the prognosis in locally advanced gastric cancer is poor and five-year survival rates are only around 20–30% following surgery [2]. Recurrence rates following surgical resection are high, especially within the first five years after surgery [3]. Even when the disease is amenable to surgical resection, multimodal treatment strategies are required to achieve optimal curative rates.

Adjuvant chemotherapy (ACT) after surgery was historically the standard of care. After much debate, the efficacy of this approach has been demonstrated in four major trials; however, standard adjuvant treatment varies regionally. In Asia, the ACTS-GC and CLASSIC trials have shown its efficacy in improving overall survival (OS) and disease-free survival (DFS) [4,5], while in North America, postoperative chemoradiotherapy is preferred by the US intergroup study and perioperative treatment by the MAGIC study in Europe [6,7]. However, postoperative complications and delay in wound healing may cause a delay in adjuvant treatment, leading to interest in alternative approaches such as neoadjuvant treatment.

The neoadjuvant treatment aims to downstage tumors, eradicate micrometastatic disease early, and increase R0 resection rates. The MAGIC and FLOT4 trials have shown that perioperative chemotherapy improves survival outcomes compared to surgery alone [7,8]. Furthermore, neoadjuvant chemotherapy (NACT) increases treatment compliance as it is administered prior to surgery. However, in contrast, if neoadjuvant chemotherapy is not effective on specific tumor biology, it carries the risk of disease progression during treatment; therefore, disease restaging should be considered [9].

Despite these benefits, the optimal sequencing of chemotherapy—neoadjuvant versus adjuvant—remains a matter of ongoing debate. Direct comparisons are challenging due to variations in chemotherapy regimens, patient-specific factors, and regional treatment preferences. The purpose of this study is to assess the oncologic and clinical results of adjuvant and neoadjuvant treatments for gastric cancer.

## 2. Materials and Methods

### 2.1. Patient Selection

This retrospective study included 103 patients diagnosed with non-metastatic gastric cancer between 2014 and 2024. Of these, 47 patients received neoadjuvant chemotherapy, while 56 underwent adjuvant chemotherapy.

The inclusion criteria were histologically confirmed non-metastatic gastric cancer, age ≥ 18 years, receipt of either neoadjuvant or adjuvant chemotherapy with curative intent, and availability of complete clinical, pathological, and follow-up data.

The exclusion criteria were presence of distant metastasis at diagnosis or missing follow-up data.

Treatment decisions (NACT vs. ACT) were made by multidisciplinary tumor boards, considering tumor location, clinical stage, performance status, and comorbidities.

During the earlier years of the study period (2014–2017), adjuvant chemotherapy following upfront surgery was the more commonly recommended approach, in line with national guidelines and prevailing clinical practice in Turkey at that time. As evidence—particularly from the FLOT4 trial—began to shift global treatment standards, neoadjuvant chemotherapy was increasingly adopted at our institution from 2018 onward.

Clinical factors influencing the decision to pursue neoadjuvant chemotherapy included suspected serosal invasion on imaging (cT3–T4), clinically positive lymph nodes, and adequate performance status (ECOG 0–1). Conversely, patients with lower-stage disease, borderline performance status, or a diagnosis made before 2018 were more likely to undergo upfront surgery followed by adjuvant chemotherapy. These evolving patterns reflect real-world clinical judgment and the progressive adoption of updated guidelines over the study period.

Treatment intolerance in the NACT group was defined as grade ≥ 3 chemotherapy-related toxicities—such as heart failure, severe gastrointestinal complications, or febrile neutropenia—that prevented patients from continuing systemic therapy or undergoing surgery.

A small number of patients lacked HER2 or PD-L1 data; these variables were therefore excluded from regression analyses.

Overall survival (OS) was defined as the time from initial diagnosis to death from any cause.

Progression-free survival (PFS) was defined as the time from initial diagnosis to either radiologically confirmed disease progression or death, whichever occurred first.

For the neoadjuvant cohort, survival analyses (both OS and PFS) included all patients who initiated neoadjuvant chemotherapy, regardless of whether they ultimately underwent surgery.

The study was approved by the Ethics Committee of Antalya Training and Research Hospital (Approval No: 10/1, Date: 20 January 2025).

### 2.2. Statistical Methods

Data were analyzed using IBM SPSS Statistics version 26 (IBM Corp., Antalya, Türkiye. Released 2019). Normality assumptions were assessed using the Kolmogorov–Smirnov and Shapiro–Wilk tests. For comparisons between groups, the independent *t*-test was used for normally distributed variables, while the Mann–Whitney U test was applied for non-normally distributed variables. Categorical variables were compared using Pearson’s chi-square test, Fisher’s exact test, or Yates’ correction, as appropriate. Multiple comparisons were conducted using the Bonferroni-adjusted Z test. Relationships between demographic/clinical features and progression or survival times were assessed using Kaplan–Meier survival analysis. Factors influencing progression and survival times were evaluated through Cox regression models. The results were presented as mean ± standard deviation or median (minimum–maximum) for continuous variables and as frequency (percentage) for categorical variables. A significance level of *p* < 0.05 was considered statistically significant.

## 3. Results

**Figure 1:**
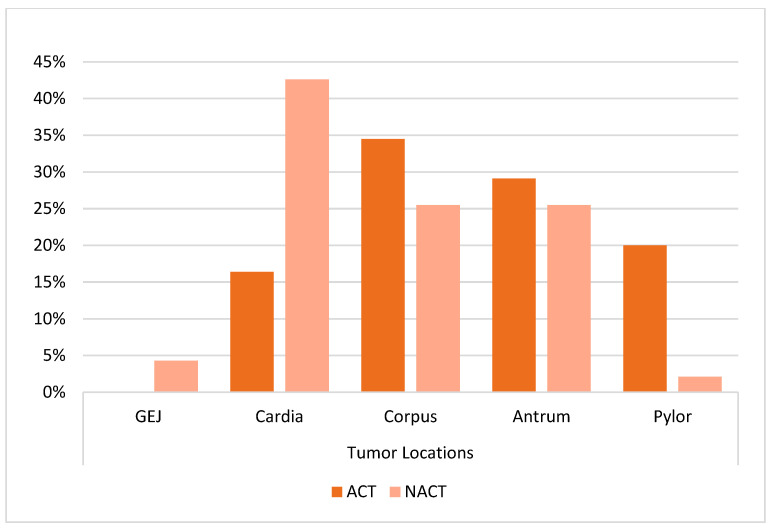
Distribution of tumor locations according to treatment groups.

As shown in Table 1, the NACT group was younger, with a mean age of 60.5 years compared to 61.8 years in the ACT group. The majority of patients in both groups were male (69.6% and 80.9%, respectively). In terms of tumor location, the NACT group had a higher prevalence of cardia tumors, while the ACT group had a higher frequency of pyloric cancers (*p* = 0.001) (Figure 1).

Nearly all patients in the NACT group presented with clinically positive lymph nodes at diagnosis (97.8% vs. 72% in the ACT group). According to clinical staging, 70% of patients in the NACT group had stage III disease, predominantly characterized by T3N1–2 tumors. In terms of pathological staging, stage III disease was more frequently observed in the ACT group (53.6% vs. 31.4% in the NACT group) (*p* = 0.030). And pathological T4 (pT4) tumors were more prevalent in the ACT group, whereas pT1 were more common in the NACT group (*p* = 0.002).

Lymphovascular invasion (LVI) and perineural invasion (PNI) were significantly higher in the ACT group. No statistically significant differences were observed between the groups in other demographic and clinical characteristics.

**Figure 2:**
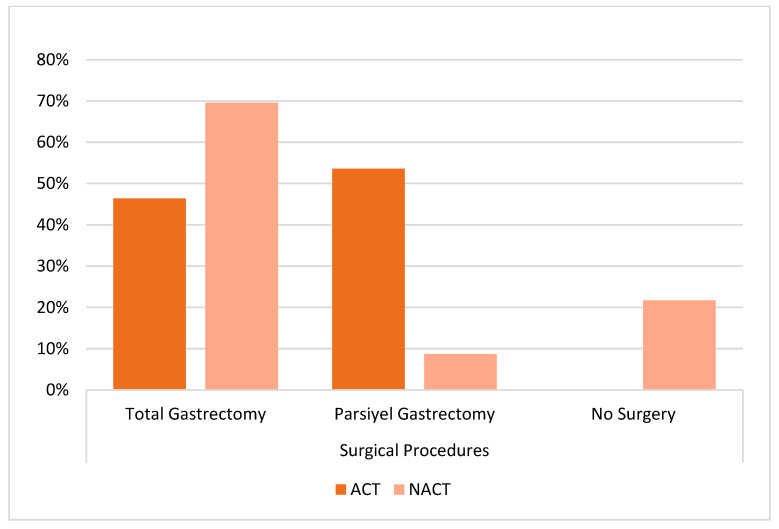
Distribution of surgical procedures according to treatment group.

In Table 2, total gastrectomy was performed more frequently in the NACT group, whereas subtotal gastrectomy was more common in the ACT group (*p* < 0.001). In the NACT group, 21% of patients did not undergo surgery. Among these, three patients declined surgery, two patients experienced chemotherapy-related adverse events (e.g., heart failure, death), and five patients had disease progression that precluded surgical intervention. D2 dissection and mean number of lymph node dissections were similar. The rate of positive surgical margins was numerically higher in the ACT group (Figure 2).

In the NACT group, patients received a mean of 4.38 treatment cycles, whereas those in the ACT group completed an average of 6.8 cycles. Notably, approximately one-third of patients in the neoadjuvant group were unable to proceed with postoperative therapy due to decreased treatment tolerance (Table 3).

**Figure 3:**
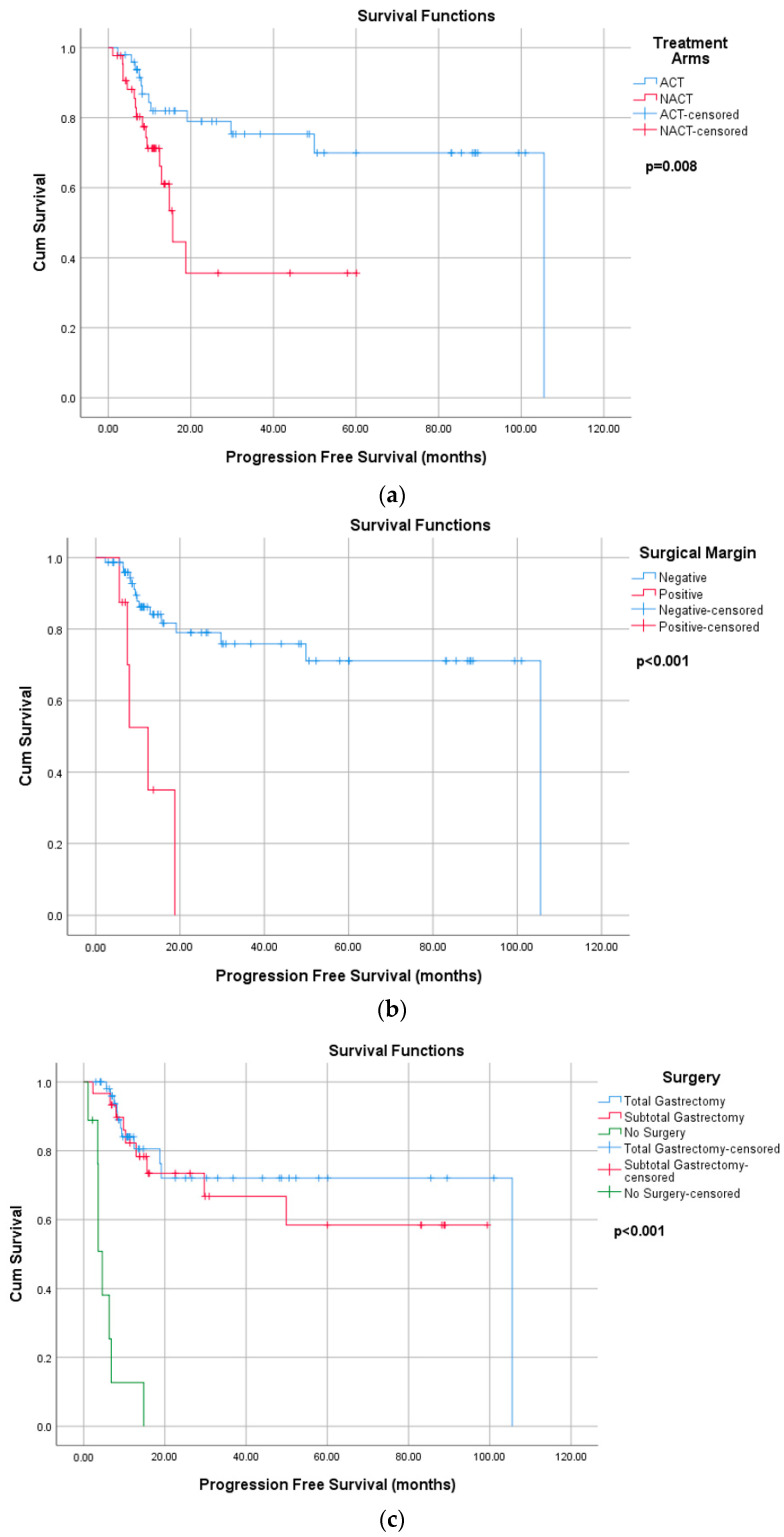
Progression free survival according to (**a**) treatment arms, (**b**) surgical margin and (**c**) surgery type. ACT: adjuvant chemotherapy; NACT: neoadjuvant chemotherapy.

According the Kaplan Maier analyses, median PFS was not reached (NR) in the ACT arm, compared to 15.6 months in the NACT arm (*p* = 0.008). The median PFS was NR in patients with negative surgical margins and 12.4 months in patients with positive surgical margins (*p* < 0.001). A statistically significant difference in PFS was also observed among patients who underwent total gastrectomy, subtotal gastrectomy, or did not undergo surgery (*p* < 0.001): the median PFS was NR for both total and subtotal gastrectomy and 4.6 months for no surgery. There was no statistically significant association between PFS and nodal status, histology, LVI, PNI, gender, or D2 dissection (Figure 3).

**Figure 4:**
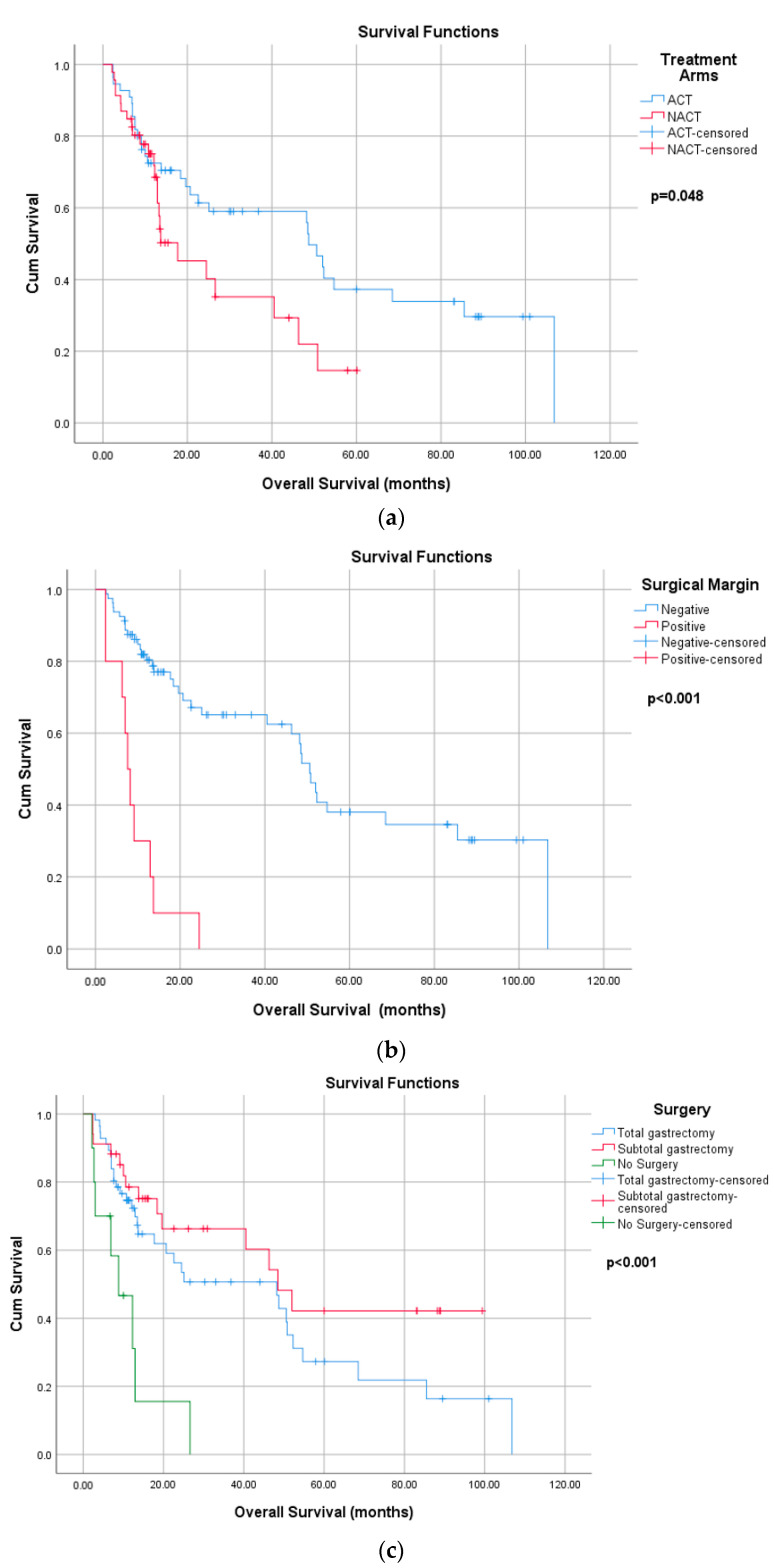
Overall survival according to (**a**) treatment arms, (**b**) surgical margin and (**c**) surgery type.

The median OS was 48.7 months in the ACT group compared to 17.7 months in the NACT group (*p* = 0.048). Patients with negative surgical margins had a median OS of 50.6 months, whereas patients with positive margins had a significantly lower median OS of 7.6 months (*p* < 0.001). A statistically significant difference in OS was also observed according to the type of surgery (48.2 months, 48.5 months, and 8.8 months, *p* < 0.001). However, no statistically significant association was found between OS and histology, nodal status, LVI, PNI, gender, or D2 dissection (Supplementary Table S1) (Figure 4).

As shown in Table 4, in univariate analysis, patients in the NACT group demonstrated a significantly higher risk of progression compared with those in the ACT group (HR: 2.8, *p* = 0.011). Patients with positive surgical margins and who did not undergo surgery exhibited a markedly increased risk of progression (*p* < 0.001). The effects of age, tumor location, and the number of lymph node dissection were not statistically significant.

In the multivariate analysis, positive surgical margins and total gastrectomy were found to be independent prognostic factors for disease progression. Patients with positive surgical margins had a significantly higher risk of disease progression (HR = 8.5, *p* < 0.001), whereas those who underwent total gastrectomy had a significantly lower risk compared to patients who did not undergo surgery (HR = 0.2, *p* = 0.023). In contrast, tumor location and treatment modality (adjuvant vs. neoadjuvant) were not significantly associated with progression (*p* > 0.05).

In the univariate analysis, age, positive surgical margins, and not undergoing surgery were significantly associated with poorer overall survival (*p* < 0.001). In contrast, tumors located in the pylorus were associated with better OS compared to cardia tumors (*p* = 0.017). In contrast, treatment modality (neoadjuvant vs. adjuvant) and the number of dissected lymph nodes did not show a significant impact on OS. In the multivariate analysis, age and positive surgical margins remained independent negative prognostic factors for OS (*p* < 0.001), whereas surgical type and tumor location did not demonstrate a statistically significant association with survival (Table 5).

## 4. Discussion

In this single-center, real-world study, we described survival outcomes and associated clinicopathological factors among patients treated with either neoadjuvant or adjuvant chemotherapy. Patients in the NACT group were younger on average, while gender distribution was comparable between the two groups. However, significant differences were observed in tumor location and pathological stage. Cardia tumors were more frequent in the NACT group, whereas pyloric tumors predominated in the ACT group.

Proximal tumor location has been recognized as an independent adverse prognostic factor in gastric cancer [10,11]. This unfavorable prognosis is often attributed to diagnosis at a more advanced stage, older patient age, and the increased need for extensive surgical resection. Moreover, postoperative mortality is generally higher in this group compared with patients with distal gastric cancer [10,12,13]. Notably, Pacelli et al. reported a significantly higher postoperative mortality rate in patients with proximal gastric tumors compared with those with distal tumors (9.6% vs. 5%, *p* = 0.033), suggesting that tumor location and surgical complexity may contribute to poorer outcomes, regardless of treatment sequencing [10]. In our study, the NACT group had a higher prevalence of proximal tumor location, a majority diagnosed at clinical stage III (node-positive), and a higher frequency of total gastrectomy, all of which likely influenced the observed survival differences. Additionally, the postoperative mortality rate in this group was 6.3%, which may have contributed to the less favorable outcomes observed.

In univariate analysis, tumors located in the pylorus demonstrated significantly better OS compared with cardia tumors, which served as the reference category. However, this statistical significance did not persist in the multivariate analysis. This finding may reflect the influence of other covariates, such as surgical margin status and age, which can have stronger prognostic effects and potentially obscure the independent role of tumor location. No significant association was found between tumor location and PFS in either univariate or multivariate analyses. These results suggest that, although tumor location is clinically relevant, its prognostic impact may be mediated or confounded by other variables. Consequently, tumor location should be considered a potential residual confounder, particularly in light of the imbalance in its distribution between treatment groups.

In the pathological evaluation, pT4 tumors and stage III disease were more frequent in the ACT group. In contrast, at clinical staging, nearly all patients in the NACT group were node-positive at diagnosis, reflecting the effective tumor downstaging achieved through preoperative therapy.

In our study, 21% of patients in the NACT group did not proceed to surgery due to disease progression, chemotherapy-related toxicity, or patient refusal. Notably, five patients experienced radiological progression during NACT. This rate is slightly higher than those reported in the literature [14]. Several studies have highlighted the prognostic implications of failing to complete multimodal therapy in resectable gastric cancer. Kakish et al. observed that approximately 15% of patients receiving NACT failed to reach surgery, highlighting the potential limitations of preoperative systemic treatment in real-world settings [14]. A follow-up analysis by Kronenfeld et al., using a large U.S. safety-net collaborative database, confirmed that attrition during NACT is a major barrier to treatment success, particularly among vulnerable patients. In this study, a markedly lower proportion of patients who experienced attrition during NACT proceeded to curative intent surgery compared to those who completed treatment (39% vs. 89%; *p* < 0.001). Moreover, among patients who underwent surgical exploration, treatment discontinuation during NACT remained an independent predictor of poorer OS (*p* = 0.004) [15]. Collectively, these findings are consistent with our observation and suggest that the inability to complete planned surgical resection may play a critical role in the poorer prognosis observed in the NACT cohort. This attrition introduces potential survivorship bias when comparing all NACT patients with only those ACT patients who completed surgery, likely favoring the outcomes of the ACT cohort.

The rates of pathological complete response (pCR) with NACT were reported to be quite low in the MAGIC and FNCLCC/FFCD trials (0% and 3%, respectively), which aligns with the 4% pCR rate observed in our study. Furthermore, in both trials, patients continued with the same chemotherapy regimens postoperatively despite the lack of pathological response in most cases, and no data support selecting a different postoperative regimen in such cases [7,16,17]. This suggests that neoadjuvant (perioperative) chemotherapy may not fully meet its expected benefits.

In gastric cancer, younger age has been identified as a poor prognostic characteristic. While younger patients are generally expected to better tolerate intensive therapy, emerging evidence suggests that early-onset gastric cancer (EOGC) may represent a biologically distinct entity with more aggressive features [18,19]. Previous studies have shown that EOGC is frequently associated with diffuse-type histology, CDH1 gene mutations, poor tumor differentiation, and advanced stage disease at diagnosis [19]. Moreover, a large population-based study reported that EOGC is associated with poor response to standard fluoropyrimidine- and platinum-based chemotherapy regimens, as well as reduced overall survival [20]. In our study, although the age difference between groups was not statistically significant, patients in the NACT group were numerically younger. These biological features may partly account for the worse outcomes observed in this cohort. Nonetheless, such interpretations should be made with caution, and further research is needed to better understand the prognostic implications of younger age in this setting.

The Kaplan–Meier analysis demonstrated longer median OS in the ACT group compared to the NACT group, although these differences must be interpreted in the context of baseline imbalances and survivorship bias. The literature indicates that adjuvant chemotherapy improves survival outcomes compared to surgery alone, as evidenced in the ACTS-GC (5-year OS: 71.7% vs. 61.1%) and CLASSIC (3-year DFS: 74% vs. 59%) trials. Conversely, the MAGIC (5-year OS: 36% vs. 23%) and FNCLCC/FFCD (5-year OS: 38% vs. 24%) trials demonstrated the survival superiority of neoadjuvant (perioperative) chemotherapy over surgery alone. Nonetheless, no direct comparative analysis of NACT and ACT has been performed to date. Although various studies have demonstrated the efficacy of multiple treatment approaches in gastric cancer, a universally accepted standard of care has yet to be established. Questions remain regarding the optimal timing of chemotherapy, the role of radiotherapy, the minimum necessary extent of lymphadenectomy, and the best chemotherapy regimen [4,5,7,17,21,22].

In our study, patients with negative surgical margins had significantly better PFS and OS compared to those with positive margins. These results align with the previous literature underscoring the role of complete tumor resection and negative margin status in improving survival outcomes in gastric cancer [23,24].

Interestingly, our analysis did not reveal significant associations between OS or PFS and certain established prognostic factors, such as lymph node involvement, LVI, PNI, histologic subtype, D2 dissection, or type of surgery. Although total gastrectomy conferred a PFS advantage compared to no surgery, this difference was not observed for OS. This finding contrasts with the existing literature, which consistently identifies LVI and PNI as markers of aggressive disease, signet ring cell histology as a predictor of poor response to chemotherapy and lower survival, and D2 dissection as essential for gastric cancer treatment [25,26,27,28]. Several factors may account for these discrepancies, including the limited sample size, variability in real-world pathology reporting, and the dominant prognostic influence of surgical margin status and treatment completion, which may have masked more subtle effects.

### Limitations

Our study has several important limitations. First, baseline imbalances existed between the treatment groups, particularly regarding tumor location, clinical and pathological staging, and other pathological features. While both clinical and pathological staging were available for patients in the neoadjuvant group, only pathological staging was recorded for those in the adjuvant group. This inconsistency is methodologically important, as neoadjuvant therapy often results in pathological downstaging, potentially confounding direct survival comparisons. Given the inability to match disease burden at diagnosis between cohorts, the observed survival differences should not be interpreted as definitive evidence of treatment superiority, but rather as preliminary findings that likely reflect baseline imbalances and real-world treatment patterns.

Second, the long study period (2014–2024) spans a decade of significant advancements in systemic therapy for gastric cancer. These temporal changes may have influenced treatment decisions, surgical approaches, and patient outcomes, contributing to clinical heterogeneity and limiting the generalizability of our findings.

Third, the retrospective design, small sample size, and baseline imbalances restricted our ability to perform comprehensive confounder adjustment. Variables were selectively included in multivariate models based on statistical significance rather than clinical relevance, which may have introduced bias and affected the robustness of our conclusions.

Fourth, the lack of a significant prognostic effect of proximal tumor location in our model, despite its well-established association with poorer outcomes, may be attributable to limited sample size or residual confounding. Tumor location may therefore have contributed to outcome differences between treatment groups.

Finally, survivorship bias is an inherent limitation of our study design. While the NACT group included all patients who initiated chemotherapy, the ACT group comprised only those who underwent and completed surgery. This design excludes patients with early progression, severe toxicity, or surgical ineligibility from the adjuvant cohort, potentially biasing survival comparisons in its favor.

## 5. Conclusions

In this real-world, single-center study, adjuvant chemotherapy was associated with longer observed OS and PFS compared with neoadjuvant chemotherapy in locally advanced gastric cancer. However, these differences are most likely explained by baseline imbalances, patient selection factors, and survivorship bias rather than the timing of chemotherapy alone. The shorter observed survival in the NACT cohort may be related to younger age, higher prevalence of proximal tumor location, more advanced stage at diagnosis, and higher rates of total gastrectomy. Additionally, the substantial proportion of patients who failed to proceed to surgery after NACT underscores a critical challenge in its clinical application.

Our findings highlight the complexity of treatment selection in gastric cancer and the limitations inherent to retrospective analyses, particularly those with baseline imbalances and evolving systemic therapies over time. While neoadjuvant chemotherapy theoretically offers advantages in tumor downstaging and early systemic control, its real-world effectiveness may be compromised by patient attrition and heterogeneity.

Therefore, these results should be interpreted with caution and primarily considered hypothesis-generating. Our study contributes to the existing literature by describing real-world treatment patterns and outcomes, rather than establishing causal superiority. Prospective, randomized controlled trials with balanced patient groups are urgently needed to define the optimal sequencing of chemotherapy and to clarify the roles of neoadjuvant versus adjuvant strategies in improving long-term outcomes for gastric cancer patients.

## Figures and Tables

**Table 1:** Demographic and clinical characteristics by groups.

Characteristic	ACT Group (*n* = 56)	NACT Group (*n* = 47)	Test Statistic	*p*-Value
**Age**	61.86 ± 12.7	60.55 ± 11.17	0.548	0.585 ^t^
**Gender**				
Female	17 (30.4)	9 (19.1)	1.701	0.192 ^x^
Male	39 (69.6)	38 (80.9)		
**Comorbidities**				
None	31 (55.4)	27 (57.4)	0.045	0.831 ^x^
Present	25 (44.6)	20 (42.6)		
**Comorbidities**				
Diabetes mellitus (DM)	10 (40)	9 (45)	4.425	0.817 ^x^
Hypertension (HT)	12 (48)	9 (45)		
Coronary artery disease (CAD)	3 (12)	5 (25)		
COPD/asthma	3 (12)	2 (10)		
Stroke/Parkinson’s disease	1 (4)	-		
Malignancy	1 (4)	1 (5)		
Dementia	1 (4)	-		
Hepatitis B (HBV)	-	1 (5)		
**ECOG PS**				
0	17 (30.4)	12 (25.5)	2.382	0.304 ^x^
1	31 (55.4)	32 (68.1)		
2	8 (14.3)	3 (6.4)		
**Histology**				
Adenocarcinoma	42 (75)	37 (78.7)	0.198	0.656 ^x^
Signet ring cell	14 (25)	10 (21.3)		
**Tumor location**				
GEJ	-	2 (4.3)	16.03	**0.001 ^f^**
Cardia	9 (16.4) ^a^	20 (42.6) ^b^		
Corpus	19 (34.5) ^a^	12 (25.5) ^a^		
Antrum	16 (29.1) ^a^	12 (25.5) ^a^		
Pyloric	11 (20) ^a^	1 (2.1) ^b^		
**Grade**				
1	4 (7.7)	1 (2.4)	1.332	0.511 ^f^
2	24 (46.2)	18 (43.9)		
3	24 (46.2)	22 (53.7)		
**Pathological stage**				
0	-	2 (5.7)	7.988	**0.030 ^f^**
1	4 (7.1) ^a^	7 (20) ^a^		
2	22 (39.3) ^a^	15 (42.9) ^a^		
3	30 (53.6) ^a^	11 (31.4) ^b^		
**Clinical stage**				
1	-	1 (2.2)	-	
2	-	11 (23.9)		
3	-	32 (69.6)		
4	-	2 (4.3)		
**cT**				
2	-	12 (26.1)	-	
3	-	25 (54.3)		
4	-	9 (19.6)		
**cN**				
0	-	1 (2.2)	-	
1	-	18 (40)		
2	-	16 (35.6)		
3	-	10 (22.2)		
**pT/ypT**				
0	-	2 (5.7)	14.570	**0.002 ^f^**
1	2 (3.6) ^a^	7 (20) ^b^		
2	3 (5.4) ^a^	5 (14.3) ^a^		
3	30 (53.6) ^a^	16 (45.7) ^a^		
4	21 (37.5) ^a^	5 (14.3) ^b^		
**pN/ypN**				
0	15 (26.8)	14 (40)	4.280	0.233 ^x^
1	13 (23.2)	5 (14.3)		
2	11 (19.6)	10 (28.6)		
3	17 (30.4)	6 (17.1)		
**Cerbb2**				
Negative	35 (68.6)	34 (79.1)	1.449	0.558 ^f^
Positive	5 (9.8)	2 (4.7)		
Not Evaluated	11 (21.6)	7 (16.3)		
**Pdl-1**				
Negative	2 (5)	7 (17.9)		
Positive	1 (2.5)	1 (2.6)		
Not Evaluated	37 (92.5)	31 (79.5)		
**LVI**				
Negative	10 (19.2)	14 (41.2)	4.921	**0.027 ^x^**
Positive	42 (80.8)	20 (58.8)		
**PNI**				
Negative	13 (24.5)	18 (52.9)	7.290	**0.007 ^x^**
Positive	40 (75.5)	16 (47.1)		

**Notes**: ^t^: independent *t*-test, ^x^: Pearson’s chi-square test, ^f^: Fisher’s exact test; ^a, b^: there is no difference between groups with same letter (Z test with Bonferroni correction), mean ± s. deviation, n (%). ACT: adjuvant chemotherapy; NACT: neoadjuvant chemotherapy cT: clinical tumor size; cN: clinical lymph node; pT: pathological tumor size; ypT: pathologic tumor size after neoadjuvant therapy; pN: pathological lymph node; ypN: pathologic lymph node after neoadjuvant therapy; PDL-1: programmed death-ligand-1; LVI: lymphovascular invasion; PNI: perineural invasion; cerbb2: human epidermal growth factor receptor 2.

**Table 2:** Comparison of surgical features according to groups.

	ACT Group (*n* =56)	NACT Group (*n* =47)	Test Statistic	*p*-Value
**Surgery**				
Total gastrectomy	26 (46.4) ^a^	32 (69.6) ^b^	32.44	**<0.001 ^f^**
Subtotal gastrectomy	30 (53.6) ^a^	4 (8.7) ^b^		
No Surgery	-	10 (21.7)		
**Surgical margin**				
Negative	49 (87.5)	33 (91.7)	-	0.735 ^f^
Positive	7 (12.5)	3 (8.3)		
**D2 dissection**				
Yes	45 (80.4)	27 (75)	0.370	0.543 ^x^
No	11 (19.6)	9 (25)		
**Number of lymph node dissection**	23 (0–70)	20 (3–62)	-0.765	0.445 ^m^

^m^: Mann–Whitney U test, ^x^: Pearson’s chi-square test, ^f^: Fisher’s exact test; ^a, b^: there is no difference between groups with same letter (Z test with Bonferroni correction), median (min.–max.), n (%).

**Table 3:** Treatments and treatment response according to groups.

	ACT Group (*n* = 56)	NACT Group (*n* = 47)
**Neoadjuvant treatment**		
Flot	-	39 (83)
Folfox	-	3 (6.4)
EOX	-	1 (2.1)
Xelox	-	1 (2.1)
CF	-	1 (2.1)
mDCF	-	2 (4.3)
**Number of neoadjuvant cycles**	-	4.38 ± 1.39
**Tumor regression score**		
0	-	3 (13.6)
1	-	5 (22.7)
2	-	7 (31.8)
3	-	7 (31.8)
**Radiological response**		
CR	-	2 (4.7)
PR	-	28 (65.1)
SD	-	8 (18.6)
PD	-	5 (11.6)
**Adjuvant treatment**		
No treatment	-	11 (30.6)
Xelox	23 (41.8)	1 (2.8)
Folfox	21 (38.2)	2 (5.6)
Fufa/Capecitabin	3 (5.5)	-
mDFC	5 (9.1)	-
CRT	8 (14.5)	3 (8.3)
Flot	-	21 (58.3)
**Number of adjuvant cycles**	6.89 ±3.31	3.22 ± 1.45
**Adjuvant radiotherapy**		
No	32 (58.2)	37 (86)
Yes	23 (41.8)	6 (14)

**Mean ± s.** deviation, n (%). CR: complete response; PR: partial response; SD: stable disease; PD: progressive disease.

**Table 4:** Factors affecting PFS.

	Univariate		Multivariate	
	HR (95% CI)	*p*	HR (95% CI)	*p*
Age	0.985 (0.953–1.019)	0.377		
Treatment arms (Ref: adjuvant)	2.836 (1.269–6.335)	**0.011**	1.564 (0.478–5.120)	0.459
Number of lymph node dissection	1.013 (0.984–1.044)	0.377		
Surgery margin (Ref: negative)	7.347 (2.516–21.458)	**<0.001**	8.555 (2.581–28.359)	**<0.001**
Surgery type (Ref: no surgery)				
Total gastrectomy	0.050 (0.018–0.135)	**<0.001**	0.205 (0.052–0.808)	**0.023**
Subtotal gastrectomy	0.064 (0.023–0.182)	**<0.001**	-	-
Tumor location (Ref: cardia)			0.617 (0.307–1.239)	0.174
Corpus	1.365 (0.536–3.478)	0.514		
Antrum	0.755 (0.253–2.253)	0.615		
Pyloric	0.422 (0.087–2.039)	0.283		
GEJ	-	0.984		

HR: hazard ratio; CI: confidence interval; GEJ: gastroesophageal junction.

**Table 5:** Factors affecting OS.

	Univariate		Multivariate	
	HR (95% CI)	*p*	HR (95% CI)	*p*
**Age**	1.043 (1.017–1.068)	**0.001**	1.069 (1.034–1.105)	**<0.001**
**Treatment arms** (Ref: adjuvant)	1.761 (0.999–3.104)	0.051		
**Number of lymph node** **dissection**	0.999 (0.977–1.021)	0.92		
**Surgery margin** (Ref: negative)	6.956 (3.226–15.000)	**<0.001**	9.316 (3.769–23.024)	**<0.001**
**Surgery type** (Ref: no surgery)			0.393 (0.135–1.149)	0.088
Total gastrectomy	0.278 (0.124–0.623)	**0.002**		
Subtotal gastrectomy	0.178 (0.072–0.439)	**<0.001**		
**Tumor location** (Ref: cardia)			0.679 (0.41–1.124)	0.132
Corpus	0.772 (0.403–1.478)	0.435		
Antrum	0.612 (0.304–1.233)	0.170		
Pyloric	0.229 (0.068–0.772)	**0.017**		
GEJ	-	0.974		

HR: hazard ratio; CI: confidence interval; GEJ: gastroesophageal junction.

## Data Availability

The data that support the findings of this study are available from the corresponding author upon reasonable request.

## Supplementary Materials

The following supporting information can be downloaded at: https://www.mdpi.com/article/10.3390/curroncol32080463/s1, Table S1: Progression-Free Survival and Overall Survival by Demographic and Clinical Variables (Kaplan–Meier Analysis).
