# Supplementary material for: Impact of Treatment Modalities on Locally Advanced Gastric Cancer—Real-World Data
Source: Curr Oncol. 2025 Aug 16;32(8):463. doi: 10.3390/curroncol32080463 (PMC12384806; doi:10.3390/curroncol32080463)
Supplement: Supplementary file 1 [file curroncol-32-00463-s001.zip › curroncol-3767333-supplementary.pdf]

Table S1. Progression-Free Survival and Overall Survival by Demographic and Clinical Variables (Kaplan–Meier Analysis)

Characteristic	OS Median (95% CI)	Test Statistic*	p-value	PFS Median (95% CI)	Test Statistic*	p-value
**Gender**						
Famele	52.00 (47.376 - 56.624)	3.300	0.069	-	0.316	0.574
Male	20.70 (12.020 - 29.380)			-		
**Comorbidities**						
None	40.50 (12.249 - 68.751)	2.281	0.131	-	0.103	0.749
Present	26.60 (0.000 - 62.214)			-		
**Treatment arms**						
ACT	48.70 (44.087 - 53.313)	3.928	**0.048**	-	6.957	**0.008**
NACT	17.70 (6.030 - 29.370)			15.60 (11.724 - 19.476)		
**ECOG PS**						
0	46.30 (0.000 - 95.863)	1.026	0.599	49.90 (1.459 - 98.341)		
1	40.50 (6.526 - 74.474)			-	3.840	0.147
2	25.10 (0.000 - 50.729)			-		
**Histology**						
Adenocarcinoma	26.60 (0.000 - 56.083)	0.826	0.364	-	0.215	0.643
Signet ring cell	46.30 (0.216 - 92.384)			-		
**Cerbb2**						
Negative	25.10 (0.019 - 50.181)	4.939	0.085	-		
Positive	12.90 (0.000 - 28.041)			14.80 (0.000 - 51.550)	1.200	0.549
Not Evaluated	68.5 (8.348 - 128.652)			-		
**pN/ypN**						
0	-	4.307	0.230	-		
1	48.70 (8.728 - 88.672)			-	6.484	0.090
2	40.50 (6.435 - 74.565)			-		
3	17.70 (9.739 - 25.661)			19.10 (0.000 - 63.206)		
**LVI**						
Negative	-	2.065	0.104	-	0.021	0.884
Positive	48.20 (19.601 - 76.799)			-		
**PNI**						
Negative	46.30 (6.744 - 85.856)	1.676	0.195	-	0.298	0.585
Positive	48.20 (15.684 - 80.716)			-		
**Surgery margin**						
Negative	50.60 (46.568 - 54.632)	32.932	**<0.001**	-	18.185	**<0.001**
Positive	7.60 (5.741 - 9.459)			12.40 (6.985 - 17.815)		
**D2 disection**						
No	40.50 (0.000 - 81.779)	0.340	0.056	-	0.687	0.407
Yes	48.50 (23.502 - 73.498)			-		
**Surgery type**						
Total gastrectomy	48.20 (17.452 - 78.948)	16.875	**<0.001**	-		
Subtotal gastrectomy	48.50 (34.746 - 62.254)			-	70.101	**<0.001**
No surgery	8.80 (2.355 - 15.245)			4.6 (3.625 - 5.575)		

*Log-rank test, CI: Confidance interval.
